# Differences in sociodemographic, drug use and health characteristics between never, former and current injecting, problematic hard-drug users in the Netherlands

**DOI:** 10.1186/1477-7517-11-6

**Published:** 2014-02-13

**Authors:** Petra Havinga, Claudia van der Velden, Anouk de Gee, Agnes van der Poel

**Affiliations:** 1 Network of Infectious Diseases and Harm Reduction, Trimbos Institute, P.O. Box 725, Utrecht 3500, AS, The Netherlands

**Keywords:** Injecting drug use, Non-injecting drug use, Housing, Homelessness, Opiate use, Health, Low-threshold care

## Abstract

**Background:**

Injecting drug users are at increased risk for harmful effects compared to non-injecting drug users. Some studies have focused on differences in characteristics between these two groups (e.g., housing, overall health). However, no study has investigated the specific Dutch situation which in the last years has seen a decrease in homelessness among problematic hard-drug users and an increasing focus on physical health in low-threshold addiction care. The purpose of this study was to determine differences in sociodemographic, drug use and health characteristics between never-injecting (NIDUs), former-injecting (FIDUs) and current-injecting drug users (IDUs) and describe injecting practices.

**Methods:**

A total of 202 problematic hard-drug users (NIDU = 64; FIDU = 76; IDU = 62) were recruited from 22 low-threshold care facilities, including drug consumption rooms, methadone maintenance treatment, heroin-assisted therapy, day shelter and/or night shelter, supported housing and day activity centres. Data were collected on-site through structured face-to-face interviews.

**Results:**

Results indicate that IDUs represented a separate group of problematic hard-drug users, with distinct sociodemographic and drug use characteristics. Overall, IDUs appeared to be the group with least favourable characteristics (unstable housing/homelessness, illegal activities, polydrug use) and NIDUs appeared to have the most favourable characteristics (stable housing, help with debts, less polydrug use). The FIDU group lies somewhere in between. The three groups did not differ significantly in terms of health. Regarding injecting practices, results showed that majority of IDUs had injected drugs for over 10 years and IDUs injected heroin, cocaine, amphetamine and/or methadone in the past 6 months. Sharing syringes was not common. A quarter reported public injecting.

**Conclusions:**

Unstable housing and homelessness are related to (former) injecting drug use, and stable housing is related to never-injecting drug use. Our study suggests that the number of ‘new’ IDUs is low. However, public injecting among IDUs is not uncommon and is associated with unstable housing. This emphasizes the potential of housing projects as a component of harm reduction measures. Therefore, prevention of (risks associated with) injecting drug use and supported housing programmes for problematic hard-drug users deserve the continuous attention of policymakers and professionals in low-threshold addiction care.

## Background

It is widely recognized that injecting drug use is the most harmful route of administration [1,2]. Compared to non-injecting drug users, injecting drug users are at increased risk for vein damage [3,4], fatal and non-fatal overdoses [5-7] and the transmission of blood-borne infectious diseases mainly due to sharing injection equipment [8-22].

According to estimates based on 2008 data, there are approximately 17,700 problematic opiate users in the Netherlands [23]. The number of injectors has decreased over the past 15 years due to the high mortality rate and the low rate of initiation into injecting drug use [24,25]. Compared to other European countries, the Netherlands has (by far) the lowest percentage of injectors [26]: an estimated 7%–13% of the problematic opiate users inject drugs [23,26].

Although many studies have investigated injecting and non-injecting drug users, few have focused on differences in characteristics between these two groups. Studies comparing injectors and non-injectors have been performed in, for example, North America, Europe and Asia and have reported a variety of differences in characteristics. In terms of *sociodemographic characteristics*, some studies suggest that non-injectors are more likely to be younger [16,19,27] and female [27]. However, other studies found no significant differences between injectors and non-injectors with regard to age and gender [10,28]. Unstable housing and income from illegal activities are more likely to occur among injectors [10,19]. Studies also indicate that injectors have a lower education level and are more likely to drop out of school compared to non-injectors [16,28,29]. With regard to *drug use characteristics*, injectors tend to start using drugs at a younger age [27,28], report a longer duration of drug use [16,19,27], a higher frequency of use [10,27] and higher rates of dependence [29] compared to non-injectors. Injectors are also more likely to use drugs other than heroin, such as cocaine and/or amphetamine [19,27]. In terms of *health characteristics*, injectors more frequently report poor or fair overall health [10] and report higher levels of somatisation and anxiety symptoms [19]. However, other studies show that non-injectors are more likely to experience mental health problems than injectors [10].

As mentioned, some studies have aimed to identify differences between injectors and non-injectors. However, no study has investigated the specific situation in the Netherlands. Therefore, little is known about differences in sociodemographic, drug use and health characteristics between injectors and non-injectors in the Netherlands. Furthermore, in the last decade, two notable developments in the Netherlands make a Dutch study of interest. First, intensive efforts from the government, municipality and social care have led to a decrease in the number of homeless problematic drug users [30,31]. Studies emphasize the potentially positive impact of stable housing on drug-related health, e.g. a decrease in drug use and reduced risk of needle/syringe sharing and unprotected sex [31-34]. Second, in the Netherlands, there seems to be an increasing focus on physical health in low-threshold addiction care, illustrated by the development and use of guidelines, the implementation of HCV screening and the increased number of medical personnel working in these facilities [35-39]. Since nearly all problematic hard-drug users are clients in addiction care, this may have a positive impact on drug-related health. With these developments in mind, the present study aims to investigate key differences between never-injecting drug users (NIDUs), former-injecting drug users (FIDUs) and current-injecting drug users (IDUs) in the Netherlands. More specifically, we examine differences in sociodemographic, drug use and health characteristics between these groups. We also describe the injecting practices of IDUs because of their strong association with health outcomes.

## Methods

### Procedure

The sample consisted of 202 problematic hard-drug users and was selected using convenience sampling. Participants were recruited in low-threshold addiction care facilities which all target problematic hard-drug users, including drug consumption rooms, methadone maintenance treatment, heroin-assisted therapy, day shelter and/or night shelter, supported housing and day activity centres. These facilities reach both injecting and non-injecting problematic hard-drug users in the Netherlands (there are no facilities specifically targeting injecting drug users). Since nearly all problematic hard-drug users are clients of low-threshold care facilities (86%) [23,40] and open drug scenes seem to have disappeared [25,41], these facilities were chosen for recruitment activities. The selection of low-threshold care facilities was made with the assistance of members of the Network of Infectious Diseases and Harm Reduction (NIHR) [42], in which all 11 Dutch regional addiction care organizations are represented. Members have an adequate view of the care provided and the corresponding target group in their region. Therefore, the selection of facilities can be characterized as a good reflection of the Dutch low-threshold care facilities for problematic hard-drug users. The NIHR is coordinated by the Trimbos Institute and is financially supported by the Ministry of Health, Welfare and Sport. All approached facilities agreed to participate in the study, which resulted in 22 recruitment sites located in all 12 provinces in the Netherlands. Twelve facilities were ‘stand-alone’ services, namely supported housing (*n* = 4), methadone maintenance treatment (*n* = 4), day activity centres (*n* = 2), heroin-assisted therapy (*n* = 1) and drug consumption rooms (*n* = 1). The remaining facilities (*n* = 10) were ‘integrated’: they offer a wider range of services on the same location. These facilities consisted of different combinations of methadone maintenance treatment, heroin-assisted therapy, drug consumption rooms and day and night shelter.

Face-to-face interviews were conducted by four trained Trimbos Institute researchers who visited each facility in teams of two or three for 4–8 h. Before their visit, two posters were distributed in the facility to inform and engage drug users in general and IDUs in particular to participate in the study. Because of the low prevalence, IDUs were intentionally overrepresented in order to provide a sufficiently large number for comparison purposes. Besides that, we were especially interested in injection practices in this population. Upon arrival at the facility, the researchers started recruiting participants for the interview, in most cases, with the help of the attending staff. Clients were eligible for participation if they had a minimum age of 18 years and had a sufficient command of the Dutch language. All clients who were willing and able to participate were included. Participants gave informed consent and were assured of anonymity at the beginning of the interview. They received €5 after the interview was completed. The interview lasted 30–60 min on average.

### Instrument

Participants were interviewed using a structured questionnaire which included questions on sociodemographic background, drug use patterns and (mental) health characteristics. IDUs were asked about injection behaviour in the past 6 months.

Two questions were used to differentiate between NIDUs, FIDUs and current IDUs [43-46]: ‘Have you ever, even once, injected any drug?’ and ‘In the past 6 months, have you injected any drug?’ Participants were categorized as NIDU (never injected drugs), FIDU (have injected drugs but not in the last 6 months) or IDU (injected drugs in the past 6 months). Sociodemographic characteristics included sex, age, ethnicity (according to Statistics Netherlands (CBS): native Dutch, Western immigrants, non-Western immigrants), highest level of education completed, legal identity (passport or identification card), currently paid or volunteer work, current illegal sources of income, stable housing, current debt situation, estimated debt amount and debt help. Illegal income was defined as income generated in the past month by property crime, violent crime, drug dealing, prostitution or begging. To measure ‘stable housing’, we asked participants about their current housing status: independent housing/rented room, sheltered housing with ambulatory support, sheltered housing with 24/7 support, temporary housing (squat, shelter, staying with friends or family) or homeless. The first three categories were defined as stable housing. In terms of health characteristics^a^, we asked the participants how many times they had visited a physician for physical complaints and if they had visited a psychiatrist or another mental health care professional in the past 12 months. General health is based on a health question measured on a five-point scale about self-perceived health: ‘How is your health in general?’ [47]. The first two categories (‘very good’ or ‘good’) were classified as ‘good perceived health’. Drug use characteristics were measured by the number of days a particular drug had been used in the past 30 days, with reference to the following substances: heroin, methadone, cocaine/crack, amphetamine, non-prescribed tranquilizers, cannabis and alcohol. Respondents were divided into two categories: daily users (≥20 days/month) and non-daily users (<20 days/month). Current injection practices included age at first injection, duration of injecting, type of drugs injected, main drug injected, injection frequency, main place of injection (physical location) and risk behaviour. Based on classifications described by Harris et al. [48], injection frequency in the past 6 months was divided into three categories: low-frequency injectors (1–60 injections), less than daily injectors (>60 injections, but not on a daily basis) and daily injectors (>182 injections). Risk behaviour indicators were syringe sharing and public injecting in the past 6 months. Furthermore, we asked participants if they had ever attempted to stop injecting.

### Data analyses

Differences between NIDUs, FIDUs and IDUs in sociodemographic, drug use and health characteristics were analyzed using chi-square tests (proportions) with 95% confidence levels (CI). A *p* value less than 0.05 was considered significant. All statistical analyses were performed using Statistical Package for the Social Sciences version 19 (SPSS 19).

## Results

Of the 202 participating problematic hard-drug users, 64 (32%) were NIDUs, 76 (38%) were FIDUs and 62 (31%) were IDUs.

### Differences between NIDUs, FIDUs and IDUs

Sociodemographic and health characteristics of NIDUs, FIDUs and IDUs are presented in Table 1, and drug use characteristics are presented in Table 2. Specifics on IDUs and injecting characteristics are presented in the next paragraph. Regarding FIDUs, the mean time since participants last injected was 10.6 years ago (median 8.0 years), and the mean duration of injecting was 12.2 years (median 10.5 years).

**Table 1:** Sociodemographic and health characteristics of NIDUs, FIDUs and current IDUs

**Characteristic**	**Total**	**NIDU**	**FIDU**	**IDU**	**Test statistic *P***
	***n *** **(%)**	***n *** **(%)**	***n *** **(%)**	***n *** **(%)**	
	**202 (100)**	**64 (32)**	**76 (38)**	**62 (31)**	
Sociodemographic					
Male	164 (81)	49 (77)	62 (82)	53 (86)	0.438
Age, years					0.343
≤39	41 (20)	13 (20)	15 (20)	13 (21)	
40–49	89 (44)	27 (42)	29 (38)	33 (53)	
≥50	72 (36)	24 (38)	32 (42)	16 (26)	
Ethnicity					0.000***
Native Dutch	115 (57)	26 (41)	50 (66)	39 (63)	
Western immigrant	46 (23)	9 (14)	21 (28)	16 (26)	
Non-Western immigrant	41 (20)	29 (45)	5 (7)	7 (11)	
Possession of passport or identification card	173 (86)	55 (86)	69 (91)	49 (79)	0.146
High school completion	126 (62)	39 (61)	48 (63)	39 (63)	0.959
Stable housing	153 (76)	56 (88)	55 (72)	42 (68)	0.024*
Social welfare payment	192 (95)	61 (95)	71 (93)	60 (97)	0.660
Paid or volunteer work	84 (42)	28 (44)	29 (38)	27 (44)	0.745
Illegal income	53 (26)	10 (16)	17 (22)	26 (42)	0.003**
Debts (in euro)	145 (71)	50 (78)	51 (67)	44 (71)	0.348
0–10,000	22 (15)	8 (16)	11 (22)	3 (7)	
10,001–100,000	62 (43)	19 (38)	25 (49)	18 (41)	
100,001–500,000	49 (34)	17 (34)	11 (21)	21 (48)	
>500,000	9 (6)	4 (8)	3 (6)	2 (5)	
Unknown	3 (2)	2 (4)	1 (2)	-	
Debt help	96 (66)	36 (72)	38 (75)	22 (50)	0.024*
Health					
Good perceived health	86 (43)	27 (42)	33 (43)	26 (42)	0.982
Visit physician for physical complaints (past 12 months)	136 (67)	44 (69)	50 (66)	42 (68)	0.930
1–4	88 (65)	24 (56)	33 (66)	31 (74)	0.271
5–10	29 (22)	11 (26)	9 (18)	9 (21)	
>10	18 (13)	8 (19)	8 (16)	2 (5)	
Visit psychiatrist/other mental health care professional (past 12 months)	70 (35)	19 (30)	26 (34)	25 (40)	0.453

*p ≤ 0.05, **p ≤ 0.01, ***p ≤ 0.001.

**Table 2:** Drug use in the past month of NIDUs, FIDUs and current IDUs

**Drug**	**Total**	**NIDU**	**FIDU**	**IDU**	**Test statistic *P***
	***n *** **(%)**	***n *** **(%)**	***n *** **(%)**	***n *** **(%)**	
	**202 (100)**	**64 (32)**	**76(38)**	**62 (31)**	
Methadone	172 (85)	44 (69)	71 (93)	57 (92)	0.000***
Cocaine	149 (74)	45 (70)	53 (70)	51 (82)	0.188
Heroin	138 (68)	40 (63)	41 (54)	57 (92)	0.000***
Cannabis	120 (59)	33 (52)	46 (61)	41 (66)	0.242
Alcohol	116 (57)	36 (56)	36 (47)	44 (71)	0.020*
Non-prescribed tranquilizers	32 (16)	3 (5)	15 (20)	14 (23)	0.011*
Amphetamine	29 (14)	7 (11)	5 (7)	17 (27)	0.002**

*p ≤ 0.05, **p ≤ 0.01, ***p ≤ 0.001.

#### Sociodemographic characteristics

NIDUs were less likely to be native Dutch than FIDUs and IDUs (41%, 66% and 63%, respectively) and were more likely to be non-Western immigrants (45%, 7% and 11%, respectively). Furthermore, a significantly higher percentage of NIDUs had stable housing compared to FIDUs and IDUs (88%, 72% and 68%, respectively). IDUs were more likely to generate income from illegal activities than FIDUs and NIDUs (42%, 22% and 16%, respectively), particularly from drug dealing, property crime and begging. Although no significant differences were found in the proportion of respondents who had debts and the amount of debts, significantly less IDUs reported that they had help with debts compared with FIDUs and NIDUs (50%, 75% and 72%, respectively).

#### Health characteristics

There were no significant differences between the three groups in terms of health characteristics.

#### Drug use characteristics

Proportionally, more IDUs reported heroin use in the past month compared to FIDUs and NIDUs (92%, 54% and 63%, respectively). The same applies to alcohol use (71%, 47% and 56%, respectively) and amphetamine use (27%, 7% and 11%, respectively). Compared to FIDUs and IDUs, NIDUs were less likely to use methadone (93%, 92% and 69%, respectively) and non-prescribed tranquilizers (20%, 23% and 5%, respectively). Methadone was used 28.8 days on average in the past month; alcohol, 18.2 days; cannabis, 18.0 days; heroin, 17.5 days; cocaine, 14.1 days; non-prescribed tranquilizers, 13.0 days; and amphetamine, 11.7 days. No significant differences were found between the three groups in the proportion of non-daily and daily users (<20 and ≥20 days per month) for each substance. The respondents used a mean of 3.7 (of the seven mentioned) substances in the past month (median 4 substances), showing that, along with the number of days that substances were used, respondents can be categorized as polydrug users.

### Current injection practices of IDUs

Current injection practices of IDUs are presented in Table 3. Among IDUs, age at first injection ranged from 13 to 58 years. About half of the sample (45%) had first injected before the age of 20; 31%, between 20 and 29 years; 11%, between 30 and 39 years; and 13%, above age 40 years. The majority have been injecting drugs for more than 10 years (76%) and had, at least once, attempted to stop injecting (84%). Five IDUs have been injecting drugs for 2 years or less. Almost all IDUs (97%) reported injecting heroin in the past 6 months, followed by cocaine (60%), amphetamine (29%) and methadone (18%). Heroin was by far the main drug injected (60%), and more than half of the sample (61%) indicated their home as the main place where injecting took place. Syringe sharing was not common (10%). In the past 6 months, 24% of the IDUs reported public injecting. With regard to injection frequency, 48% of IDUs were low-frequency injectors, 18% were less than daily injectors and 32% injected on a daily basis.

**Table 3:** **Current injection characteristics of IDUs (*n*** **= 62)**

**Current injection characteristics**	***n *** **(%)**
Injection practices	
Age (in years) at first injection	
13–19	28 (45)
20–29	19 (31)
30–39	7 (11)
≥40	8 (13)
Duration of injecting	
0–2 years	5 (8)
3–9 years	10 (16)
10–19 years	14 (23)
≥ 20 years	33 (53)
Drug injected (in past 6 months)	
Heroin	60 (97)
Cocaine	37 (60)
Amphetamine	18 (29)
Methadone	11 (18)
Main drug injected (in past 6 months)	
Heroin	37 (60)
Cocaine	14 (23)
Amphetamine	7 (11)
Other	4 (7)
Frequency of injecting (in past 6 months)	
Low frequency (1–60 injections)	30 (48)
Less than daily (61–182 injections)	11 (18)
Daily (>182 injections)	20 (32)
Main place of injection (in past 6 months)	
Home	38 (61)
Drug Consumption Room	11 (18)
Friends home	8 (13)
Other facilities (e.g. HAT)	3 (5)
Public places	2 (3)
Attempt(s) to stop injecting	52 (84)
Risk behaviour	
Syringe sharing (in past 6 months)	6 (10)
Public injecting (in past 6 months)	15 (24)

## Discussion

This study investigated the differences between NIDUs, FIDUs and IDUs in the Netherlands, with a special focus on IDUs due to their increased risk of harm.

### Differences between NIDUs, FIDUs and IDUs

Our findings indicate that IDUs represent a separate group of problematic hard-drug users, with distinct sociodemographic and drug use characteristics. Overall, IDUs appear to be the group with the least favourable sociodemographic and drug use characteristics, the NIDU group has the most favourable characteristics and the FIDUs fall somewhere in between. However, the three groups did not differ in terms of health characteristics. Results show that NIDUs are more likely than FIDUs and IDUs to be non-Western immigrants. A possible explanation is that injecting drug use may—at least in part—be inhibited by cultural factors and that other conditions (e.g. social cohesion, being distinctive as a group from ‘white junkie injectors’ [49]) were favourable for the popularity of the chasing ritual [1,49]. Another finding is that unstable housing is more frequent among IDUs. This is in agreement with previous studies suggesting a link between injecting drug use and bad housing status or homelessness [10,33,50-53]. We found that IDUs are more likely to generate income from illegal activities such as drug dealing, crime against property and begging. Other studies reported similar results [10,19]. While no difference was found between the groups with respect to debts, the results did show that only half of the IDUs receive help with debts as compared to about 75% in the other groups. This finding is difficult to explain. Additional analyses showed that problematic hard-drug users with unstable housing are less likely to receive debt help compared to those with stable housing (45% and 74%, respectively; *p* = 0.001). However, being a NIDU, FIDU or IDU does not seem to be meaningful within this context. IDUs seem to have more unfavourable drug use characteristics compared to NIDUs and FIDUs. Although there were no substantial differences between the average days of drug use, significantly, more IDUs report the use of heroin, alcohol and amphetamine. Polydrug use is common among IDUs, as evidenced by the high proportions of IDUs that used various drugs in the last month (e.g. methadone 92%, cocaine 82%, heroin 92%). This is in line with others who found that proportionally more drug users who identified injecting as their main route of heroin use declared to have (ever) used more than one drug in addition to heroin [19,27], e.g. cocaine and benzodiazepines. The three groups showed no differences in terms of health characteristics, yet previous studies did identify health differences [10,19]. These inconsistencies in findings may be due to differences in classifications used to distinguish between injectors and non-injectors. For instance, Fischer et al. [10] found that significantly more current non-injectors (i.e. those who had not injected in the last 30 days) had good overall health compared to current injectors (i.e. those who had injected in the last 30 days) and that mental health problems were more frequent among current non-injectors. In contrast, Stohler et al. [19] reported higher levels of somatisation and anxiety symptoms in heroin users who predominantly injected compared to heroin users for whom chasing was the preferred and most used route of heroin administration. Furthermore, this result might be explained by the fact that, in the Netherlands, almost all problematic hard-drug users, injectors as well as non-injectors, are clients of low-threshold care facilities [23,40] in which the focus on health has increased in recent years [37-39].

### Injecting practices

Our findings suggest that the number of problematic hard-drug users who recently initiated injecting is low. The IDU group in our study mainly consists of long-term injectors. More than 75% of the IDUs have been injecting drugs for ≥10 years, while only five IDUs are so-called ‘new injectors’, i.e. those injecting for ≤2 years [54]. This is in line with other research showing a continuing decline in prevalence rates of IDUs over the last decades [24-26].

Research in the Netherlands showed that amphetamine users hardly choose injection as route of administration [55]. In our study, approximately 30% of the IDUs had injected amphetamine in the past 6 months which indicates that amphetamine injection is not uncommon among IDUs. As these studies took different approaches (respectively amphetamine users and IDUs), it is not clear if our result suggests an increased popularity of injection as a route of administering amphetamine. Future research should examine the prevalence of amphetamine injecting and identify motives for injecting. Understanding the motives for injecting amphetamine may contribute to the development of effective preventive interventions.

Because syringe or needle sharing is not common among IDUs (10% in the past 6 months), the risk of transmission of infectious diseases through sharing appears to be relatively small. This proportion seems to be in line with previous estimates based on 16 surveys reporting prevalence rates of 11%–30% of IDUs who had borrowed syringes or needles from someone else [56]. Broad availability of needle and syringe exchange programmes (NSP) from which IDUs can obtain injecting equipment easily and for no or low cost probably played a substantial role in reducing syringe sharing. A rough estimation suggests that, currently, there are about 150 NSPs in the Netherlands [57]. Supported housing programmes and drug consumption rooms have probably contributed to the reduction of risks associated with injecting in public spaces [33,58]. However, our results clearly indicate that public injecting is still a topic of serious concern. About 25% of the IDUs in our sample reported public injecting in the past 6 months. There is evidence linking unstable housing/homelessness to injecting in public spaces [34,53,59,60]. Additional analyses showed that a higher proportion of IDUs living in unstable housing conditions reported injecting in public places compared to IDUs living in stable housing conditions (40% and 17%, respectively). Although results did not reach statistical significance (*p* = 0.06), housing may be a factor that positively influences injecting practices, including public injecting.

### Limitations

Some limitations of the study should be considered. First, generalizability of the results may be limited due to the overrepresentation of IDUs in our sample for comparison purposes. The results of the total group are not representative of problematic hard-drug users in general, in contrast to the results concerning the subgroups of IDUs, FIDUs and NIDUs. Subgroup results were confirmed by the members of the NIHR, which support the generalizability of these results. Second, self-reported data are used. The validity and reliability of such data are considered to be questionable, although several studies reported behavioural self-reports of problematic hard-drug users to be sufficiently reliable and of good quality [61-63]. Finally, the cross-sectional study design does not allow drawing conclusions about causality.

## Conclusions

This study was the first to investigate differences in characteristics between NIDUs, FIDUS and IDUs in the Netherlands, indicating that IDUs seem to be the group with the least favourable characteristics (e.g. unstable housing, illegal activities, no debt help). The large majority of problematic hard-drug users—both injectors and non-injectors—make use of low-threshold addiction care facilities where respondents were interviewed. Increased attention for physical and mental health within these facilities has resulted in harm reduction taking place at different levels, as the facilities themselves as well as the interventions within these facilities aim to reduce harm, including improvements in health. Present findings on public injecting and debt help support previous research which emphasizes the potential of housing projects as a component of harm reduction measures. Therefore, it is important to continue housing policy measures for example within the strategy plans for social relief of all of the 43 so-called centre municipalities that cover the Netherlands. Access to housing for problematic hard-drug users is part of this policy. It is worth mentioning that residents of neighbourhoods where sheltered housing for problematic hard-drug users are located often feared public nuisance and crime. Local research, especially in the city of Utrecht where sheltered housing started in the Netherlands, showed that this fear is largely ungrounded [31]. Last, it is recommended to put intensive efforts into explicitly and directly targeting IDUs. Although injecting is on the decline, injecting drug use and public injecting still exist and remain a threat to public health. Prevention of injecting and of risks associated with injecting fits in with the increasing attention for physical health in the Dutch low-threshold addiction care and remains a topic of serious interest.

## Endnotes

^a^We collected the self-reported status of hepatitis B, hepatitis C and HIV of the respondents; we are currently comparing these self-reported data to registered data within addiction care. The results are the topic of a paper in preparation.

## Competing interests

The authors declare that they have no competing interests.

## Authors’ contributions

PH and AP designed the study. PH and CV carried out the data collection. PH and AP were responsible for data analysis, interpretation of the data and drafting the manuscript. CV and AG made contributions. All authors read and approved the final manuscript.
